# Direct Reprogramming of Adult Human Somatic Stem Cells Into Functional Neurons Using *Sox2, Ascl1*, and *Neurog2*

**DOI:** 10.3389/fncel.2018.00155

**Published:** 2018-06-08

**Authors:** Jessica Alves de Medeiros Araújo, Markus M. Hilscher, Diego Marques-Coelho, Daiane C. F. Golbert, Deborah A. Cornelio, Silvia R. Batistuzzo de Medeiros, Richardson N. Leão, Marcos R. Costa

**Affiliations:** ^1^Brain Institute, Federal University of Rio Grande do Norte, Natal, Brazil; ^2^Bioinformatics Multidisciplinary Environment, IMD, Federal University of Rio Grande do Norte, Natal, Brazil; ^3^Laboratório de Biologia Molecular e Genômica, Centro de Biociências, Federal University of Rio Grande do Norte, Natal, Brazil

**Keywords:** induced neurons, lineage reprogramming, human mesenchymal stem cells, umbilical cord, proneural genes

## Abstract

Reprogramming of somatic cells into induced pluripotent stem cells (iPS) or directly into cells from a different lineage, including neurons, has revolutionized research in regenerative medicine in recent years. Mesenchymal stem cells are good candidates for lineage reprogramming and autologous transplantation, since they can be easily isolated from accessible sources in adult humans, such as bone marrow and dental tissues. Here, we demonstrate that expression of the transcription factors (TFs) SRY (sex determining region Y)-box 2 (*Sox2*), Mammalian achaete-scute homolog 1 (*Ascl1*), or Neurogenin 2 (*Neurog2*) is sufficient for reprogramming human umbilical cord mesenchymal stem cells (hUCMSC) into induced neurons (iNs). Furthermore, the combination of *Sox2/Ascl1* or *Sox2/Neurog2* is sufficient to reprogram up to 50% of transfected hUCMSCs into iNs showing electrical properties of mature neurons and establishing synaptic contacts with co-culture primary neurons. Finally, we show evidence supporting the notion that different combinations of TFs (*Sox2/Ascl1* and *Sox2/Neurog2*) may induce multiple and overlapping neuronal phenotypes in lineage-reprogrammed iNs, suggesting that neuronal fate is determined by a combination of signals involving the TFs used for reprogramming but also the internal state of the converted cell. Altogether, the data presented here contribute to the advancement of techniques aiming at obtaining specific neuronal phenotypes from lineage-converted human somatic cells to treat neurological disorders.

## Introduction

Reprogramming of somatic cells into induced pluripotent stem cells (iPS) that can generate all three major embryonic lineages, stem cells and even a new animal has revolutionized research in regenerative medicine in recent years (Takahashi and Yamanaka, 2006; Okita et al., 2007). Somatic cells isolated from different sources can be converted into iPS (Meissner et al., 2007; Aoi et al., 2008; Hanna et al., 2008; Espejel et al., 2010; Imamura et al., 2010), which in turn can be converted into specific cell types including neurons (Wernig et al., 2008; Kuzmenkin et al., 2009; Mizuno et al., 2010; Zhang et al., 2013). However, generation of iPS and further differentiation into neuronal cells is time consuming and the cells retain tumorigenic potential (Takahashi and Yamanaka, 2006; Okita et al., 2007).

In contrast, direct lineage reprogramming of somatic cells is a fast process and bypasses the pluripotent stage associated with tumor transformation. Astrocytes isolated from the postnatal cerebral cortex of mice were the first cells to be directly reprogrammed into induced neurons (iNs) following expression of the transcription factor (TF) Neurogenin 2 (*Neurog2*) or Mammalian achaete-scute homolog 1 (*Mash1/Ascl1*) (Berninger et al., 2007; Heinrich et al., 2011). Subsequently, the list of murine cell types liable to lineage reprogramming into iNs grew substantially, including non-neural cells, such as mouse fibroblasts and hepatocytes (Vierbuchen et al., 2010; Marro et al., 2011). However, non-neural cells typically require more than one TF to achieve a full neuronal conversion.

Direct reprogramming of human somatic cells into neurons can also be achieved through expression of *Ascl1* in combination with other TFs (Ambasudhan et al., 2011; Son et al., 2011; Karow et al., 2012). It has also been reported that expression of *Ascl1* or *Neurog2* alone is sufficient to induce conversion of human fibroblasts into induced neurons (Chanda et al., 2014; Gascón et al., 2016), but the efficiency of this process is low (< 10%). Moreover, the phenotypes of iNs obtained through direct cell lineage reprogramming using human cells remains largely elusive. Pinpointing strategies capable of producing iNs exhibiting defined neurochemical phenotypes is a critical step towards translation of the lineage reprogramming techniques into clinics.

Here, we show that the expression of the transcription factor SRY (sex determining region Y)-box 2 (*Sox2*), *Ascl1* or *Neurog2* is sufficient to lineage-convert a small fraction of human umbilical cord mesenchymal stem cells (hUCMSCs) into iNs. In contrast, the co-expression of either *Sox2/Ascl1* or *Sox2/Neurog2* is sufficient to convert a large fraction of hUCMSCs (up to 50%) into iNs displaying electrophysiological hallmarks of mature neurons and establishing synaptic contacts with other cells. Furthermore, we show that iNs may express transcripts associated with the acquisition of different neurochemical phenotypes, independently of the combination of transcription factors used. Also, *Sox2/Ascl1* and *Sox2/Neurog2* may induce the expression of genes involved in the acquisition of the same neurochemical phenotypes, suggesting that iNs fate during lineage-conversion is influenced by other aspects than the transcription factors used. Collectively, our data indicate that hUCMSCs are good candidates for lineage reprogramming into iNs, but more studies are required to further advance protocols capable of producing iNs with a particular phenotype.

## Materials and methods

### Cell culture

Human multipotent mesenchymal stem cells (hMSC) were isolated from umbilical cords donated with informed consent of the pregnant mothers at maternity Januário Cicco, Federal University of Rio Grande do Norte, Natal, Brazil. The study was approved by the Research Ethics Committee of the Federal University of Rio Grande do Norte (Project Number 508.459), and in strict agreement with Brazilian law (Resolution 196/96). All subjects gave written informed consent in accordance with the Declaration of Helsinki.

In this study, Wharton's jelly mesenchymal stem cells were isolated from umbilical cord. Following isolation from the subendothelium vein, according to the method previously published (Duarte et al., 2012), the remaining umbilical cord tissue was cut in small pieces and washed with phosphate-buffered saline (PBS; 137 mM NaCl, 2.7 mM KCl, 4.3 mM Na_2_HPO_4_, and 1.47 mM KH_2_PO_4_; Merck), supplemented with 3% antibiotic–antimycotic solution (prepared with 10,000 units/ml penicillin G sodium, 10,000 μg/ml streptomycin sulfate and 25 μg/ml amphotericin B; HyClone). Then, the tissue was centrifuged at 200 g for 10 min, and the pellet resuspended in 10 mL of 0.1% collagenase type IV (Worthington) diluted in PBS. After that, the explants were incubated for 16 h at 37°C in a water bath. The tissue was centrifuged again at 200 g for 10 min, the pellet washed twice with PBS and then gently dissociated in a digestion solution containing 0.25% trypsin and 0.02% EDTA (Invitrogen) for 15 min at room temperature. To interrupt trypsin activity, we added fetal bovine serum (FBS; HyClone). Once again, the cell suspension was centrifuged, and the cell pellet resuspended in minimum essential medium a (α MEM; Gibco Invitrogen) supplemented with 10% FBS and 1% antibiotic solution. Cells were plated onto T25 tissue culture flasks (TPP) and these cultures maintained at 37°C in a humidified atmosphere containing 5% CO_2_. After 2 or 4 days, the medium was changed and non-adherent cells were removed. Cultures consisting of small, adherent and spindle shaped fibroblastoid cells reaching 60–70% of confluence were detached and subcultured at 4,000 cells/cm^2^.

### Characterization of hMSCs

The cells isolated from Wharton's jelly human umbilical cord were characterized as MSCs, according to the criteria proposed by the International Society for Cellular Therapy (Horwitz et al., 2005; Dominici et al., 2006). The hMSCs were labeled with a panel of monoclonal antibodies against several cell markers, including CD105-FITC, CD90PE-Cy5 (Bioscience), CD73PE, CD34PE, HLA-DR-FITC, CD45-FITC, and CD14PE (Becton Dickinson's). Briefly, the cells were detached of the tissue culture plates using 0.25% trypsin/EDTA, washed, and homogenized with PBS. They were then incubated with monoclonal antibody for 30 min in darkness at room temperature. At the end of this period, the cell suspension was centrifuged, washed in PBS, and re-suspended in cold fixing solution, 0.5% formaldehyde in PBS. For each test, isotype-matched monoclonal antibodies were used as negative controls (IgG1-FITC, PE, and PE-Cy5; Becton Dickinson's). The fluorescence intensity of labeled cells was determined with a fluorescence-activated cell analyzer (FACScan) using cell quest software (Cell Quest™ Software, Becton Dickinson Immunocytometry Systems), a total of 20,000 events for each sample were recorded. The following parameters were considered: forward scatter in linear scale (which evaluates cell size), side scatter in linear scale (assessing cell complexity), and cell marker expression in fluorescence analysis by FL1, FL2, and FL3 in logarithmical scale, representing the antigen–antibody reaction conjugated to FITC, PE, and PE-Cy5, respectively. Results were expressed as a percentage of cells labeled with monoclonal antibodies. Osteogenic, adipogenic, and chondrogenic differentiation assays were carried out according to methodology previously published (Duarte et al., 2012).

### Plasmids

The pro-neural genes *Ascl1, Neurog2*, or *Sox2* were expressed under control of an internal chicken β-actin promoter with cytomegalovirus enhancer (pCAG) together with DsRed or GFP behind an internal ribosomal entry site (pCAG-Ascl1-IRES-DsRed, pCAG-Neurog2-IRES-DsRed, and pCAG-Sox2-IRES-GFP). For control experiments, cultures were transfected with plasmids encoding only DsRed or GFP (pCAG-IRES-DsRed or pCAG-IRES-GFP) (Heinrich et al., 2010; Karow et al., 2012). Plasmid stocks were prepared in *Escherichia coli* and purified using the endotoxin-free Maxiprep plasmid kit (Invitrogen). DNA concentration was adjusted to 1 μg/μL in TE buffer endotoxin free, and plasmids were stored at −20°C.

### Transfection

For transfections, hMSC were seeded in 24-well plates onto poly-D-lysine (Sigma-Aldrich) and laminin (L-2020; Sigma Aldrich) coated glass coverslips at a density of 3 × 10^4^ cells per well in 0.5 mL α MEM (Gibco) supplemented with 10% fetal bovine serum (FBS) and 1% antibiotic solution (penicillin/streptomycin). The cells were grown in these conditions for 1–3 days until 70–80% confluent.

Both DNA plasmids (1 μg/μL) and a lipophilic cationic reagent (Lipofectamine 2000, Invitrogen) were diluted in 50 μL Opti-MEM (Reduced Serum Medium, Invitrogen). Mixtures were incubated for 5 min and then combined for a further 20 min according to the manufacturer's instructions. Complexes were added to the cells in a total volume of 0.5 mL Opti-MEM (Gibco) and incubated at 37°C in a humidified atmosphere containing 5% CO_2_ for 10–12 h. Antibiotics and serum were not used during transfection procedures.

### Co-culture with hippocampal neurons

For co-culture experiments, mouse hippocampus at postnatal day 0 to 4 (P0-4) were dissected in ice-cold PBS and dissociated in a the digestion solution for 10 min at 37°C. Trypsin action was interrupted with fetal bovine serum and the tissue dissociated mechanically with a fire-polished glass Pasteur pipette. The suspension was then centrifuged at 200 g for 5 min and washed twice in DMEM/F12 10% FBS in DMEM/F12 medium (Gibco). Mouse hippocampal cells were added to the human cultures 1–2 days after transfection at a density of 50,000 cells per well. The local University Animal Care and Use Committee (CEUA/UFRN) approved experiments involving mice. All experiments were carried out in accordance with international guidelines and regulations for animal use.

### Immunocytochemistry

Cell cultures were fixed in 4% paraformaldehyde (PFA) in PBS for 15 min at room temperature. Primary antibodies were diluted in PBS, 0.5% Triton X-100 and 5% normal goat serum. Specimens were incubated overnight at 4°C. After three washes with PBS, cells were incubated with species-specific secondary antibodies conjugated to fluorophores for 2 h at room temperature. Once again, samples were washed with PBS three times. For nuclei staining, cells were incubated for 5 min with 0.1 μg/mL DAPI (4′6′-diamino-2-phenylindone) in PBS 0.1 M. Coverslips were finally mounted onto a glass slide with a mounting medium (Aqua Poly/Mount; Polysciences). The following primary antibodies and dilutions were used: chicken anti-Green Fluorescent Protein (GFP, Aves Labs, 1:1,000), rabbit anti-Red Fluorescent Protein (RFP, Rockland, 1:1,000), mouse anti-major microtubule associated protein (MAP2; Sigma, 1:500), guinea pig polyclonal anti-vesicular GABA transporter (vGAT, Synaptic Systems, 1:200), and polyclonal anti-vesicular glutamate transporter 1 (vGLUT11, Synaptic Systems, 1:1,000).

### Electrophysiology

Cell cultures with induced neurons were transferred to a recording chamber mounted on the stage of a microscope equipped with a water immersion 40X objective (Zeiss Examiner. A1, 1 NA) and perfused with oxygenated external solution (1–1.25 ml/min) at 37°C. Data were acquired using a patch-clamp amplifier Axopatch 200B (Molecular Devices) in current or voltage clamp mode, a 16-bit data acquisition card (National Instruments), and WinWCP or WinEDR software implemented by Dr. John Dempster (University of Strathclyde). Patch-pipettes of borosilicate glass capillaries (GC150F-10 Harvard Apparatus) were pulled on a vertical puller (Narishige) with resistances from 5–7 MΩ. Pipettes were filled with internal solution (~290 Osm) containing (in mM) 130 K^+^-gluconate, 7 NaCl, 0.1 EGTA, 0.3 MgCl_2_, 0.8 CaCl_2_, 2 Mg-ATP, 0.5 NaGTP, 10 HEPES, and 2 EGTA (pH 7.2 adjusted with KOH 1M). The external solution (~300 Osm) contained (in mM) 120 NaCl, 3 KCl, 1.2 MgCl_2_, 2.5 CaCl_2_, 23 NaHCO_3_, 5 HEPES, and 11 Glucose (pH 7.4 adjusted with NaOH 1M).

Patch-clamped cells were measured for input resistance, resting membrane potential, and capacitance. Recordings were analyzed with custom routines in MATLAB. Action potentials were triggered by 400-ms depolarizing current injections from 100 pA, 400 ms, with 10 pA increments. The first fired action potential in response to minimal current injection was analyzed for amplitude (peak to afterhyperpolarization voltage), half-width (halfway between threshold voltage and peak), and afterhyperpolarization amplitude (threshold to minimum of voltage trough between the first and the second action potential in a spike train). Instantaneous and steady-state voltage were analyzed in response to hyperpolarizing current injections (−100 pA, 400 ms). Excitatory postsynaptic currents were analyzed for amplitude and rise time in free-run traces of 150 s. Active and passive electrophysiological membrane properties, including action potential parameters were analyzed using a Student's unpaired, two-tailed *t*-test.

### Calcium imaging

Calcium imaging was performed on human MSC 3 weeks post-transfection using Oregon green 488 BAPTA-1 (Invitrogen, 10 μM). Imaging was performed in physiological saline solution containing (in mM) 140 NaCl, 5 KCl, 2 MgCl_2_, 2 CaCl_2_, 10 HEPES, 10 glucose, and 6 sucrose (pH 7.35). Images were acquired approximately every 10 ms using a scientific CMOS camera (Andor). The microscope was controlled by Micro-Manager software together with the image processor ImageJ. Changes in fluorescence were measured for individual cells and average of the first 10 time-lapse images for each region of interest (ROI) was defined as initial fluorescence (F0).

### Single cell RT-qPCR

After electrophysiological recordings, the cell was sucked into the recording pipette. Pipettes were quickly removed and broken into 1.5 mL tubes containing 20 U of RNase inhibitor and 8.3 mM DTT. Samples were frozen immediately on dry ice and stored at −80°C. Immediately after thaw, the samples were treated to eliminate contaminating DNA molecules. Complementary DNA (cDNA) synthesis and pre-amplified reactions were performed with the RT^2^ PreAMP cDNA Synthesis Kit following the manufacturer's procedure (QIAGEN). Amplification was performed on the Applied Biosystems ViiA 7 Real-Time PCR (Applied Biosystems). RT^2^ Profiler PCR Array were customized in 96-well plates, designed for analyzing the expression of the following genes: Choline O-acetyltransferase (*CHAT*), Tyrosine hydroxylase (*TH*), Tryptophan hydroxylase 2 (*TPH2*), Vesicular glutamate transporter 1 (*VGLUT1* or *SLC17A7*), GABA Vesicular transporter (*VGAT* or *SLC32A1*), FEZ family zinc finger 2 (*FEZF2*), T-box brain 1 (*TBR1*), SATB homeobox 2 (*SATB2*), COUP-TF-interacting protein 2 (*CTIP2* or *BCL11B*), Platelet-derived growth factor receptor, beta polypeptide (*PDGFRB*), Thy-1 cell surface antigen (*THY-1*), Atonal homolog 8 (*ATOH8*), Neurogenic differentiation 1 (*NEUROD1*), Glyceraldehyde-3-phosphate dehydrogenase (*GAPDH*), and Hypoxanthine phosphoribosyltransferase 1 (*HPRT1*). The RT-qPCR was performed using the RT^2^ profiler PCR customized array (QIAGEN). Each array included genomic DNA control primer set, reverse transcription control, positive PCR control to report the efficiency of the polymerase chain reaction itself, and the endogenous reference genes *GAPDH* and *HPRT1*.

### Analysis of single-cell RT-qPCR

A single-cell RT-qPCR pre-processing was performed based on method described by Ståhlberg et al. (2013). Melting curve analysis performed elimination of false positives. Next, relative quantities were calculated using a cycle of quantification cutoff (Cq-cutoff) and relative-quantities of cDNA molecule equation. Missing data were imputed with absolute value 0.5, followed by conversion to log_2_-scale. Mean center and auto scale, for each gene mean center and auto scale were calculated separately using log_2_-values. Heat map and Principal Component Analysis (PCA) were used to visualize expression differences between groups. Statistical analysis and plotting were performed using the software R version 3.3.3.

### Statistical analysis

All statistical data are presented as the mean ± standard error of the mean (SEM) of at least three independent experiments. Statistically significant differences were assessed by Student's unpaired *t*-test or one-way Analysis of variance (ANOVA), comparing two or more groups, respectively. *P* < 0.05 was considered a significant difference (^*^).

## Results

### Direct lineage reprogramming of human umbilical cord MSCs

**Figure 1:**
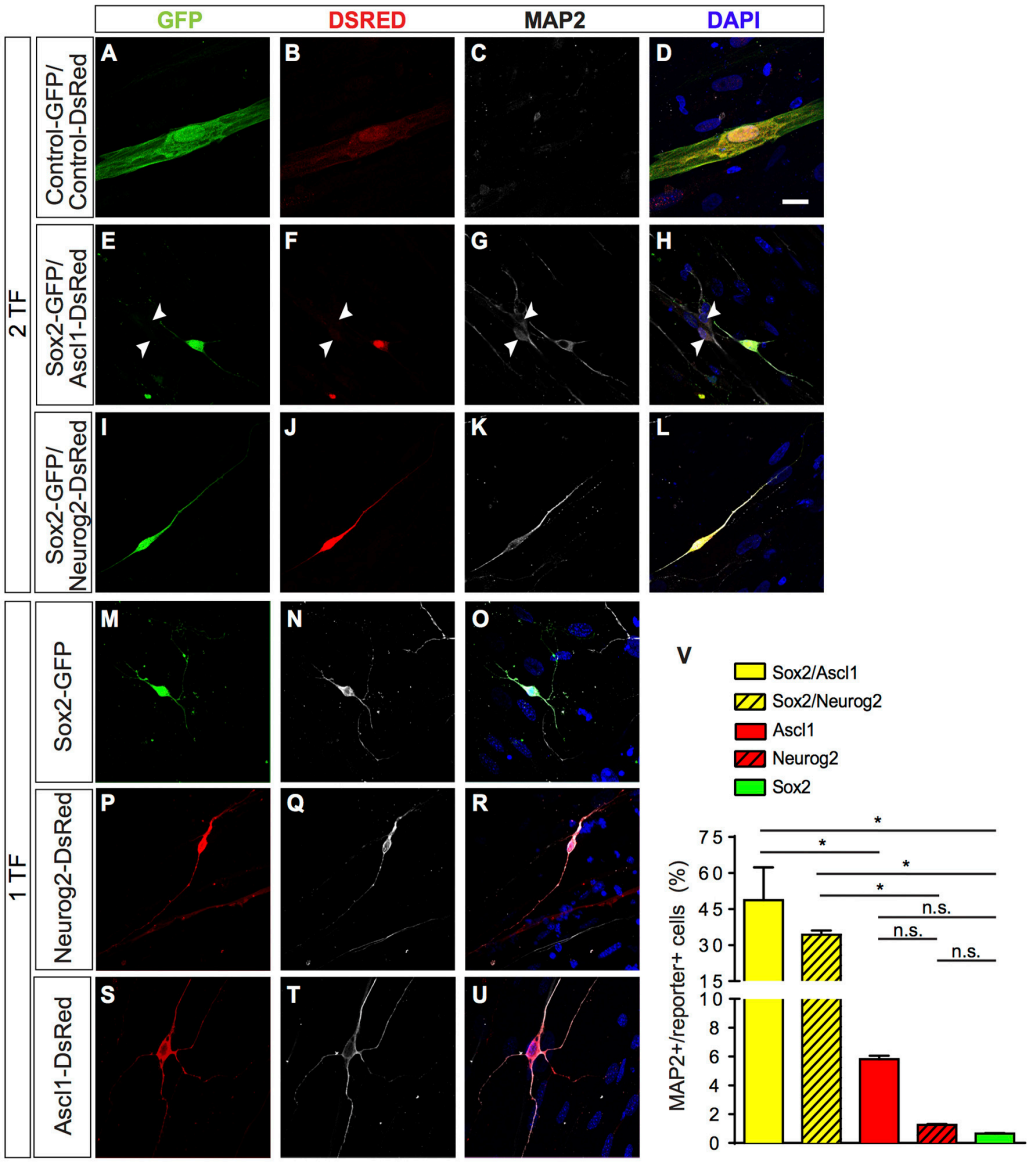
Direct lineage reprogramming of hUCMSC into iN by forced expression of *Sox2, Ascl1*, or *Neurog2* alone, *Sox2/Ascl1* or *Sox2/Neurog2*. **(A–U)** Immunostaining for DSRED (red), GFP (green), MAP2 (white), and DAPI (blue), 15 days post transfection (dpt). Scale bar represents 20μm. **(A–D)** Example of hUCMSC transfected with control plasmids encoding only reporter proteins GFP and DSRED. Note that cell displayed classical mesenchymal cell morphologies and did not express MAP2. **(E–H)** Example of hUCMSC transfected with *Sox2* and *Ascl1* (white arrows indicate hippocampal neurons expressing MAP2). **(I–L)** hUCMSC transfected with *Sox2* and *Neurog2*. **(M–O)** hUCMSC transfected with only *Sox2*. **(P–R)** hMSC transfected with only *Neurog2*. **(S–U)** hUCMSC transfected with only *Ascl1*. **(V)** Histograms show the percentage of induced neurons, measured by the number of expressing MAP2 cells over the total number of reporter positive cells. Data are presented as mean ± s.e.m. from three independent experiments. ANOVA followed by Dunn's post-hoc test, *p < 0.05; no statistically significant difference (n.s.). hUCMSC transfected with control plasmids did not express MAP2, thereby the bar is not being shown.

Mesenchymal stem cells (MSCs) can be isolated from different sources in adult humans, including the bone marrow and umbilical cord (Ding et al., 2011). These cells are highly plastic, retaining the potential to generate chondroblasts, adipocytes, and osteoblasts (Caplan, 1991; Dominici et al., 2006; Afanasyev et al., 2010; Keating, 2012). In order to characterize the cells isolated from Wharton's jelly umbilical cord, we first evaluated the expression of MSC-specific antigens using flow cytometry. Virtually all cells exhibited expression of CD105, CD73, and CD90 markers, and lacked the expression of hematopoietic lineage markers, such as CD14, CD34, and CD45 (Supplementary Figures 1A–I). The MSCs also demonstrated capacity for osteogenic, adipogenic, and chondrogenic differentiation (Supplementary Figures 1J–L). Given this versatility, we hypothesized that expression of neurogenic transcription factors in MSCs could directly reprogram these cells into neurons. To test this possibility, we transfected plasmids carrying the genes encoding for *Sox2, Neurog2*, or *Ascl1* into human umbilical cord mesenchymal stem cells (hUCMSCs) using lipophilic cationic reagent. To monitor transduced cells, all vectors carried a fluorescent protein (GFP or DsRed) under control of an internal chicken β-actin promoter with cytomegalovirus enhancer (pCAG). Vectors expressing GFP or DsRed alone were used as control (Figures 1A,B). One day after transfection, cultured medium of hUCMSCs was replaced with neuronal differentiation medium containing B27. In this medium, most transfected hUCMSCs underwent cell death precluding analysis of lineage reprogramming (Supplementary Figure 2). To overcome this limitation, we co-cultured neonatal mouse hippocampal cells with hUCMSCs. We found an average of about 50% GFP+/DsRed+ hUCMSC per field 15 days after transfection and in the presence of co-cultured neonatal mouse hippocampal cells. In contrast, the average number of GFP+/DsRed+ hUCMSCs in the absence of co-cultured cells was <4% (Supplementary Figure 2). Low number of cells was also observed in Hucmsc cultures transfected with control plasmids, indicating that the cell death under these culture conditions was independent of lineage-reprogramming. It is likely that the withdrawal of serum performed after transfection (aiming at the differentiation of induced neurons) affects the survival of hUCMSCs, whereas addition of co-cultured cells, somehow, counteracts this cell-death effect. We, therefore, concluded that co-cultures are necessary to support hUCMSC in the culture conditions used.

A few days after transfection, we observed that some hUCMSCs transfected with proneural genes acquired neuronal-like morphology. To confirm this possible lineage conversion of hUCMSCs into iNs, we further analyzed the expression of the neuronal-specific microtubule-associate protein 2 (MAP2) 15 days after transfection with proneural genes (Figure 1). A fraction of cells transfected with proneural genes expressed MAP2 and acquired small-round cell bodies and thin and long processes, resembling immature neurons. Whereas, hUCMSCs transfected with control plasmids displayed classical mesenchymal cell morphologies and did not express MAP2 (Figures 1A–U). Expression of *Sox2, Ascl1*, or *Neurog2* alone was sufficient to reprogram hUCMSCs into iNs, albeit at low rates (Figure 1V). However, the combination of *Sox2* and *Ascl1* increased the efficiency of reprogramming up to 49%, whereas the combination of *Sox2* and *Neurog2* increased the efficiency up to 35% (Figure 1V). We obtained these results using Wharton's jelly mesenchymal stem cells isolated from tree different donors and did not observed any heterogeneity in the potential of reprogramming (data not shown). These data indicate that single proneural TFs have potential to elicit lineage reprogramming of hUCMSCs into iNs, but that the synergistic action of the TFs *Sox2/Ascl1* or *Sox2/Neurog2* is sufficient to induce neuronal phenotype in a high number of hUCMSCs.

**Figure 2:**
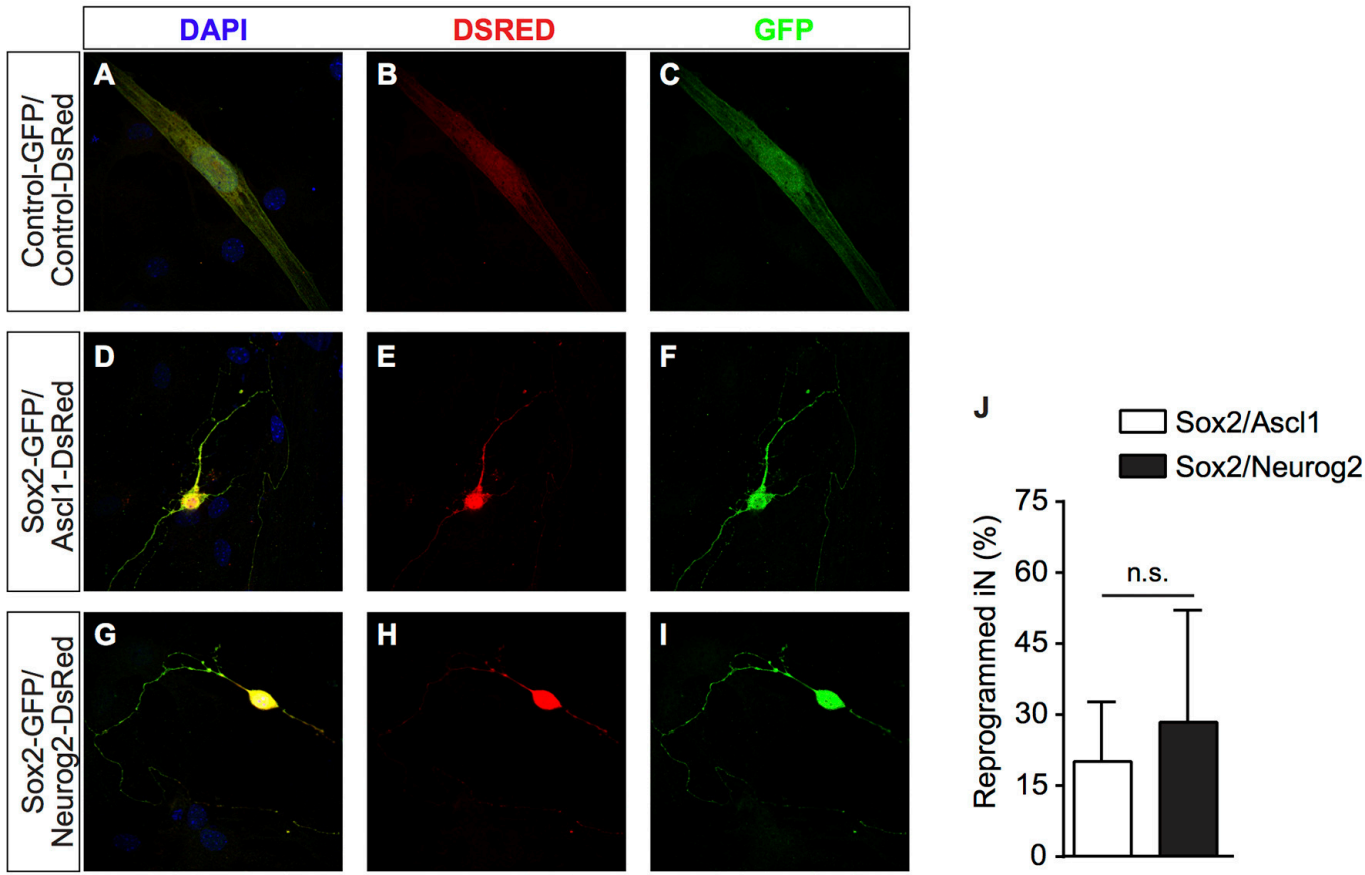
After transfection, cells were grown on astrocyte monolayers which provide support to hUCMSCs-derived iNs. **(A–C)** hUCMSCs transfected with control, **(D–F)** *Sox2/Ascl1*, or **(G–I)** *Sox2/Neurog2*. **(J)** Histogram shows the percentage of hUCMSCs-derived iNs. Data are presented as mean ± s.e.m. from three independent experiments, Student's unpaired *t*-test, no statistically significant difference (n.s.). hUCMSC transfected with control plasmids were not reprogramed into induced neurons, therefore the bar is not being shown.

It has been shown that cells from the mesenchymal lineage can fuse with other cell types in culture (Terada et al., 2002; Alvarez-Dolado et al., 2003). To rule out the possibility that hUCMSCs could be fusing with mouse hippocampal neurons present in our co-cultures, we performed similar experiments co-culturing reprogrammed hUCMSCs with purified postnatal mouse cortical astrocytes (Heinrich et al., 2011). Similar to cultures containing neonatal hippocampal cells, we observed that hUCMSCs transfected with *Sox2/Ascl1* or *Sox2/Neurog2* survived in astrocyte monolayers and acquired neuronal-like morphologies (Figures 2A–I). Thus, lineage reprogramming of hUCMSCs into iN after *Sox2/Ascl1* or *Sox2/Neurog2* expression is unlikely to be attributed to cell fusion with primary co-cultured neurons. However, we observed a lower lineage conversion efficiency when plating hUCMSCs on astrocytes (Figure 2J) compared to hippocampal cells suggesting that additional factors released by co-cultured neurons may affect either the reprogramming efficiency or survival of iNs.

### Functional properties of induced neurons

**Figure 3:**
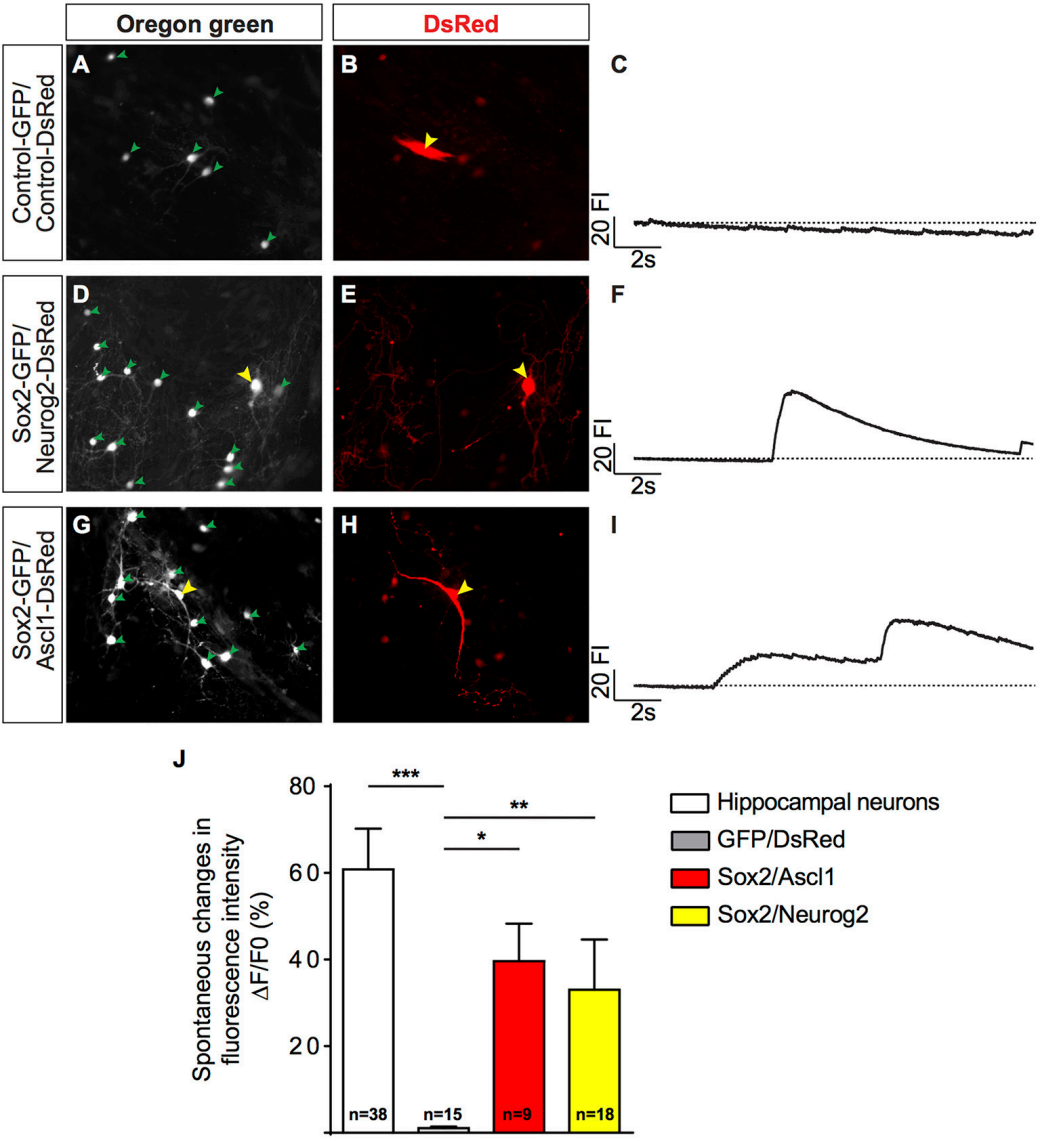
Human MSC lineage-converted iNs show fast calcium transients. **(A,B)** hUCMSCs transfected with control plasmids encoding only reporter proteins GFP and DSRED 23 dpt (yellow arrow). **(D,E)** hUCMSCs transfected with *Sox2* and *Neurog2* with neuronal morphology 23 dpt (yellow arrow). **(G,H)** Example of hUCMSCs transfected with *Sox2* and *Ascl1* with neuronal morphology 23 dpt (yellow arrow). Note the presence of mouse hippocampal neurons (green arrowheads) in the same fields. **(C,F,I)** Representative traces of time course calcium-transients of transfected hUCMSCs are shown by spontaneous variations in the fluorescence intensity (FI). **(J)** Histograms show the mean change in fluorescence of mouse hippocampal neurons and iNs. Responses were calculated as the change in fluorescence (1F) over the initial fluorescence (F0). Number of cells analyzed is indicated in the bars for each group. (ANOVA followed by Tukey's *post-hoc* test, ^*^p < 0.05; ^**^p < 0.01; ^***^p < 0.001).

To investigate if iNs could establish synaptic connections with neighboring neurons, we studied the dynamics of calcium transients using calcium sensitive dye imaging (Rosenberg and Spitzer, 2011). We measured the spontaneous changes in fluorescence intensity (ΔF/F0) during the total period of imaging (17 s) and compared their responses with primary murine hippocampal neurons. We found that both human iNs and mouse hippocampal neurons present in the co-culture displayed fast calcium-transients as indicated by rapid variations in the fluorescence (Figures 3A–I, Supplementary Movies 1, 2). The iNs reprogrammed with *Sox2/Ascl1, Sox2/Neurog2*, and the mouse hippocampal neurons showed a mean variation in fluorescence intensity of 39.66% (Figure 3J, gray bar), 33.05% (Figure 3J, blue bar), of 60.83% (Figure 3J, white bar), respectively. In contrast, the change in fluorescence intensity observed in hUCMSCs transfected with control plasmids presented a mean value of 1.1% (Figure 3J, black bar), significantly lower than the responses observed in iNs and hippocampal neurons (ANOVA followed by Tukey's post-test, *p* < 0.0001). These observations indicate that hUCMSCs-derived iNs present fast calcium transients qualitatively similar of those observed in primary neurons.

**Figure 4:**
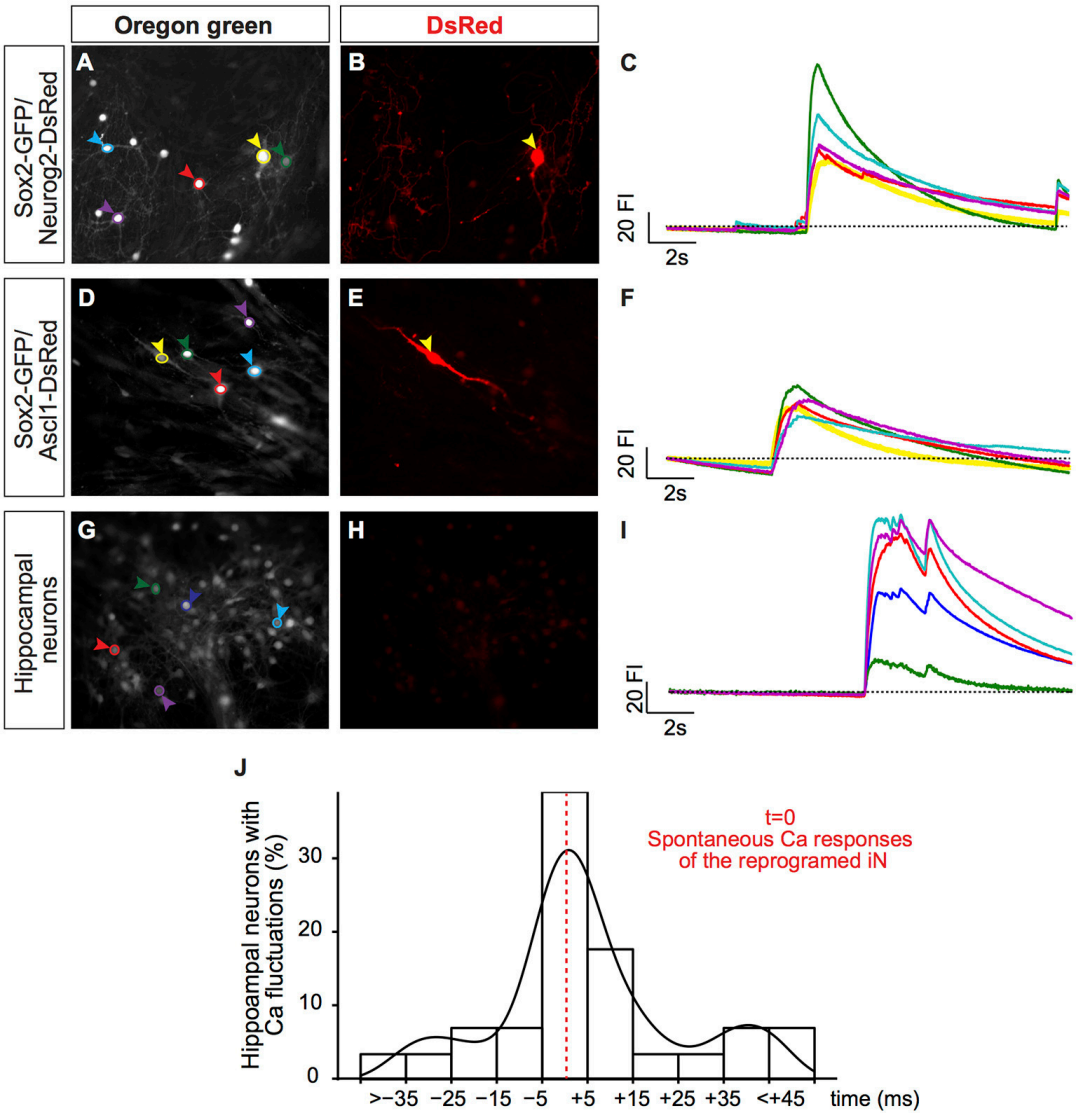
Synchronization of calcium transients between iNs and primary neurons. **(A,D,G)** Photomicrographs show hUCMSC cultures 23 dpt and labeled with BAPTA Oregon Green. Colored circles delimit the regions of interest (ROI) where fluorescence intensity (FI) was measured (colored arrowheads). **(B,E,H)** Photomicrographs of the same fields show DsRed expression. Yellow arrowheads **(A,B, D,E)** point to hUCMSC-derived iNs. Blue, red, green and purple arrowheads point to primary hippocampal neurons. **(C,F,I)** Graphics show spontaneous calcium transients. The color of ROIs in the left panel **(A,D,G)** corresponds to the color of each trace in the right panel **(C,F,I)**. The traces show spontaneous variations in the FI during 17s of recording. **(J)** Histogram shows percentage of hippocampal neurons that respond within a range time difference (ms) in the same area, *t* = 0 was considered the time of spontaneous calcium responses of the reprogrammed iN present in the same field of imaging. Density curve is represented in black.

Notably, this sudden increase in fluorescence intensity in hUCMSC-derived iNs was temporally synchronized mouse hippocampal neurons in the same field of observation (Figures 4A–I). To quantify this phenomenon, we measured the percentage of hippocampal neurons showing elevation in the fluorescence intensity within a time-range (ms) of the fluorescence fluctuation observed in a single iN within the same field of observation. The time of the iN calcium transient was considered as *t* = 0. We found that the majority of mouse hippocampal neurons showed changes in fluorescence intensity within 15 ms of fluctuations observed in iNs (Figure 4J), indicating a strong synchronization of calcium transients among primary neurons and iNs. Such a strong synchronization within the frame of milliseconds may suggest that cells are synaptically connected (Dawitz et al., 2011). To further confirm that hUCMSCs-derived iNs could receive synaptic inputs, we performed patch clamp recordings on these cells.

**Figure 5:**
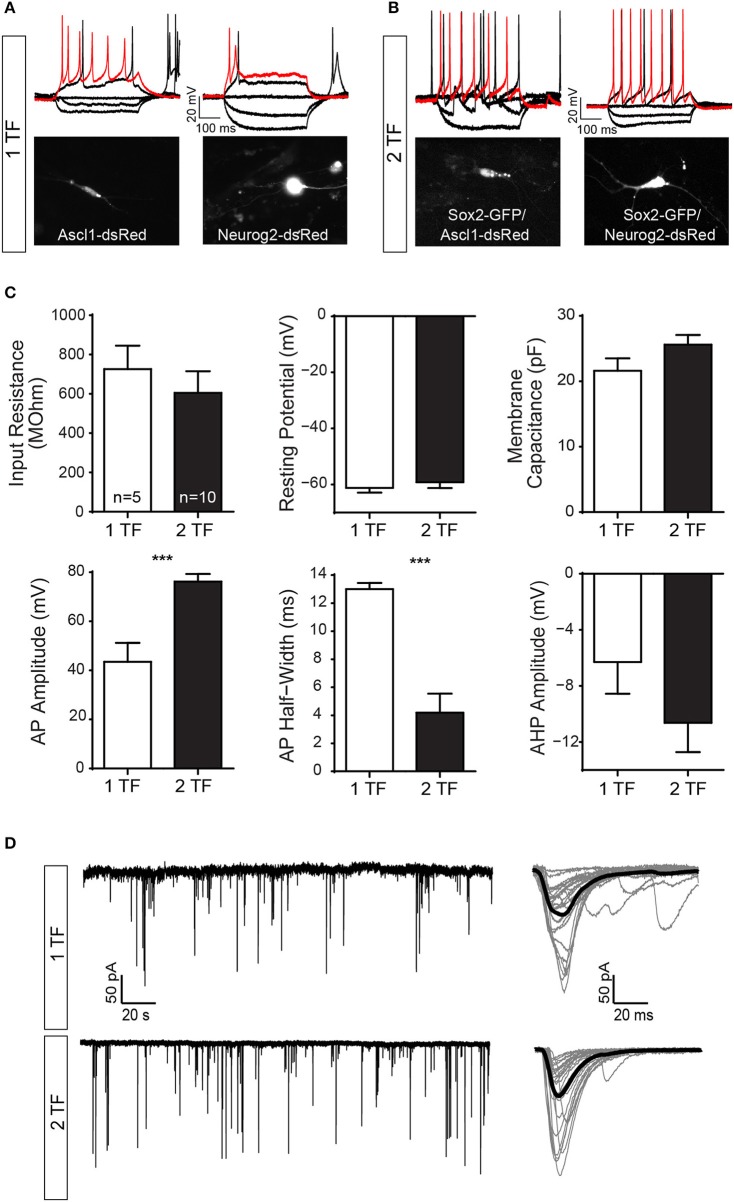
hUCMSCs-derived iNs show electrical properties of mature neurons and establish synaptic contacts with co-culture mouse primary neurons. Electrophysiological properties of cells reprogrammed with one transcription factor (1 TF) compared to cells reprogrammed with two transcription factors (2 TF). **(A)** Current clamp traces from cells with 1 TF (left: *Ascl11*; right: *Neurog2*) showing regular spiking pattern (left), and startle onset (right) in response to depolarizing current injections [50 pA (black), 100 pA (red), 400 ms]. **(B)** Example of current clamp traces from cells with 2 TF (left: *Sox2/Ascl1*; right: *Sox2/Neurog2*) responding with a regular spiking pattern (black: 50 pA; red: 100pA; 400 ms). Note that hyperpolarizing current injections caused some cells to rebound (−50 and −100 pA, 400 ms). Fluorescence images of the recorded cells are displayed below. **(C)** Bar graphs showing mean and SEM input resistance, resting membrane potential and capacitance (top) as well as mean action potential (AP) amplitude, AP half-width and afterhyperpolarization (AHP) amplitude (bottom) for cells with 1 TF (white bars) and 2 TF (black bars) respectively (Student's unpaired *t*-test, ^***^p < 0.001). **(D)** Example of voltage clamp trace (free run) showing spontaneous EPSCs of a reprogramed 1TF iN (top, left) and a 2TF iN (bottom, left). Single postsynaptic currents are plot in gray (right) and the corresponding mean trace is shown in black (*n* = 25).

We performed patch-clamp recordings on iNs reprogrammed with one transcription factor (1 TF; *n* = 5) and compared their active and passive properties to cells reprogrammed with two transcription factors (2 TF; *n* = 10). Cells with 1 TF had a mean input resistance of 726 ± 119 MΩ, resting membrane potential of −61 ± 2 mV and capacitance of 22 ± 2 pF. Out of the 5 cells, 2 responded with regular spiking pattern (Figure 5A, top left), 1 responded with startle onset (Figure 5A, top right), and 2 with a spikelet in response to depolarizing current injections (0–100 pA, 400 ms, with 10 pA increments). Spikes were analyzed for action potential amplitude, action potential half-width and afterhyperpolarization amplitude. Spikes of cells with 1 TF had a mean action potential amplitude of 44 ± 8 mV, action potential half-width of 13 ± 0.5 ms and afterhyperpolarization amplitude of −6 ± 2 mV (Figure 5C). In comparison, cells reprogrammed with 2 TF had a mean input resistance of 605 ± 110 MΩ (*p* = 0.51), resting membrane potential of −59 ± 2 mV (*p* = 0.54) and capacitance of 26 ± 1 pF (*p* = 0.13). Of the 10 cells with 2 TF, 6 responded with regular spiking pattern and 4 responded with startle onset (0–100 pA, 400 ms, with 10 pA increments). Spikes of cells with 2 TF had a mean action potential amplitude of 76 ± 3 mV (*p* = 0.0004), action potential half-width of 4 ± 1 ms (*p* = 0.0006) and afterhyperpolarization amplitude of −11 ± 2 mV (*p* = 0.22; Figure 5B). While hyperpolarizing current injections (0 to −100 pA, 400 ms, with 10 pA decrements) caused some cells to rebound (*n* = 3), prominent membrane sags could only be detected in cells transfected with *Sox2/Neurog2* (*n* = 7; Figure 5B, right) suggesting that these cells have a sizeable hyperpolarization-activated current. Comparing instantaneous and steady state voltage in response to negative current injections (−100 pA, 400 ms) showed a significant difference between instantaneous (−71 ± 2 mV) and steady state (−66 ± 1 mV) values (*p* = 0.0427).

We also observed spontaneous excitatory postsynaptic currents (EPSCs) in iN during voltage clamp (Figure 5D), suggesting that lineage-reprogrammed iNs could receive synaptic contacts from other neurons. We compared the first 100 events of 1 TF cells and 2 TF cells against each other. Postsynaptic currents of cells with 1 TF had an amplitude of 82 ± 9.7 pA and rise time of 11 ± 0.4 ms. The postsynaptic currents of cells with 2 TF had an amplitude of 86 ± 15.1 pA (*p* = 0.8312) and a mean rise time of 9 ± 0.3 ms (*p* = 0.0211).

### *Sox2/Neurog2* and *Sox2/Ascl1* induce different neuronal phenotypes in hUCMSCs

Next, we set out to evaluate the expression of messenger RNAs (mRNA) of genes commonly expressed in either hMSCs or neurons. To that, we collected single cells using a glass-micropipette, isolated the total mRNA, reverse transcribed, and pre-amplified cDNAs that were used in RT-qPCR reactions. We observed that the average expression level of common MSCs genes *THY1* and *PDGFB* was decreased in the iNs (Supplementary Figure 3A), whereas the expression of the neuronal genes *ATHO8* or *NEUROD1* increased after expression of *Sox2/Ascl1* or *Sox2/Neurog2* in hMSCs, respectively (Supplementary Figure 3B). Combined with our previous observations, these data indicate that hMSCs were effectively converted into iNs by forced expression of proneural TFs.

**Figure 6:**
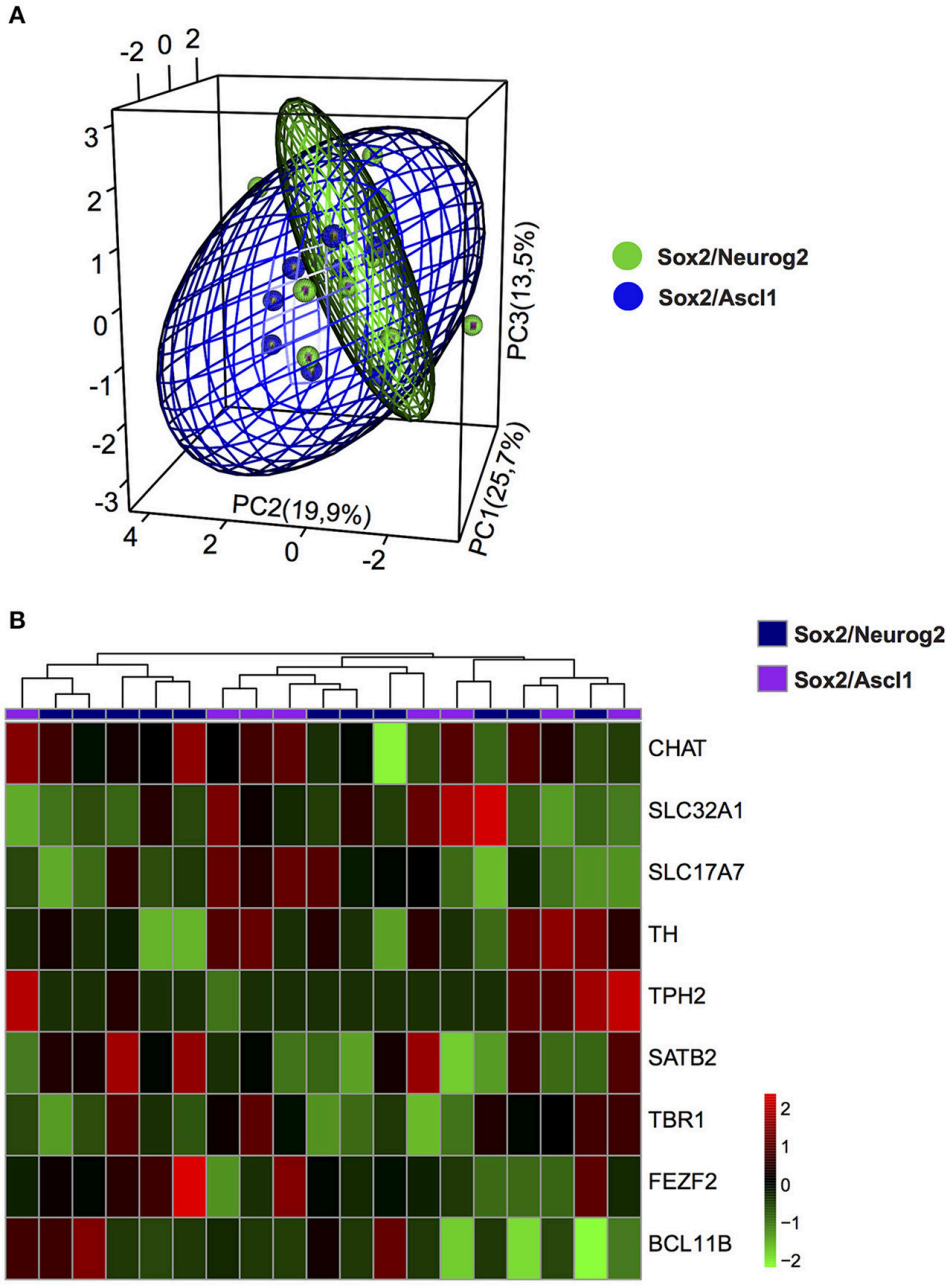
Phenotypic specification of lineage reprogrammed hUCMSC-derived iNs. **(A)** Principal component analysis (PCA) of gene expression among cells reprogrammed with *Sox2/Neurog2* or *Sox2/Ascl1*. Genes used in the PCA are involved in neurotransmitter identity. Note the significant overlap between the two cell populations, suggesting that expression of either *Sox2/Neurog2* or *Sox2/Ascl1*/ may elicit similar neuronal phenotypes. **(B)** Heat map showing the relative expression of 9 genes involved in the specification of different neuronal phenotypes. Observe the variable expression of genes essential for the specification of distinct neurotransmitter identities in iNs derived from hUCMSCs lineage-converted through the expression of either *Sox2/Neurog2* or *Sox2/Ascl1*. Choline O-acetyltransferase (CHAT), Tyrosine hydroxylase (TH), Tryptophan hydroxylase 2 (TPH2), Vesicular Glutamate Transporter 1 (VGLUT1 or SLC17A7), GABA Vesicular Transporter (VGAT or SLC32A1), FEZ family zinc finger 2 (FEZF2), T-box brain 1 (TBR1), SATB homeobox 2 (SATB2), COUP-TF-Interacting Protein 2 (CTIP2 or BCL11B).

To evaluate the possible phenotypes adopted by hMSC-derived iNs, we analysed the expression of known genes expressed by cholinergic (*CHAT*), dopaminergic (*TH*), serotoninergic (*TPH2*), glutamatergic (*SLC17A7*), and GABAergic (*SLC32A1*) neurons, as well as genes encoding for transcription factors associated with specific classes of glutamatergic neurons within the cerebral cortex (*FEZF2* and *BCL11B*—corticofugal neurons; *TBR1*—cortico-thalamic neurons; *SATB2*—callosal neurons). Relative expression of these transcripts was calculated using a cycle of quantification cutoff (Cq-cutoff) and relative-quantities of cDNA molecule equation (Ståhlberg et al., 2013). Next, we used unsupervised PCA analysis to classify iNs obtained from hUCMSCs expressing either *Sox2/Neurog2* or *Sox2/Ascl1*. We observed that the expression levels of the transcripts for *CHAT, TH, TPH2, SLC17A7, SLC32A1, FEZF2, BCL11B, TBR1*, and *SATB2* could not clearly distinguish the two populations of cells (Figure 6A), indicating that similar genes were regulated by both combinations of TFs in hUCMSCs-derived iNs. Indeed, we observed that both *Sox2/Ascl1* and *Sox2/Neurog2* could induce the expression of genes associated with distinct neurochemical phenotypes in hUCMSCs-derived iNs, although some phenotypes were more commonly observed for a given TF combination. For instance, *Sox2/Ascl1* generated more iNs expressing high levels of *TPH2*, whereas *Sox2/Neurog2* generated more *CHAT* expressing iNs. Nevertheless, the expression of all transcripts analyzed was regulated by both combinations of TFs, suggesting that *Ascl1* and *Neurog2* do not have a unique role in the phenotypic specification of lineage reprogrammed hUCMSC-derived iNs (Figure 6B). These data suggest that the expression of *Sox2/Ascl1* or *Sox2/Neurog2* in hUCMSC activates a transcriptional program associated with loss of mesenchymal phenotype and acquisition of multiple neuronal phenotypes.

**Figure 7:**
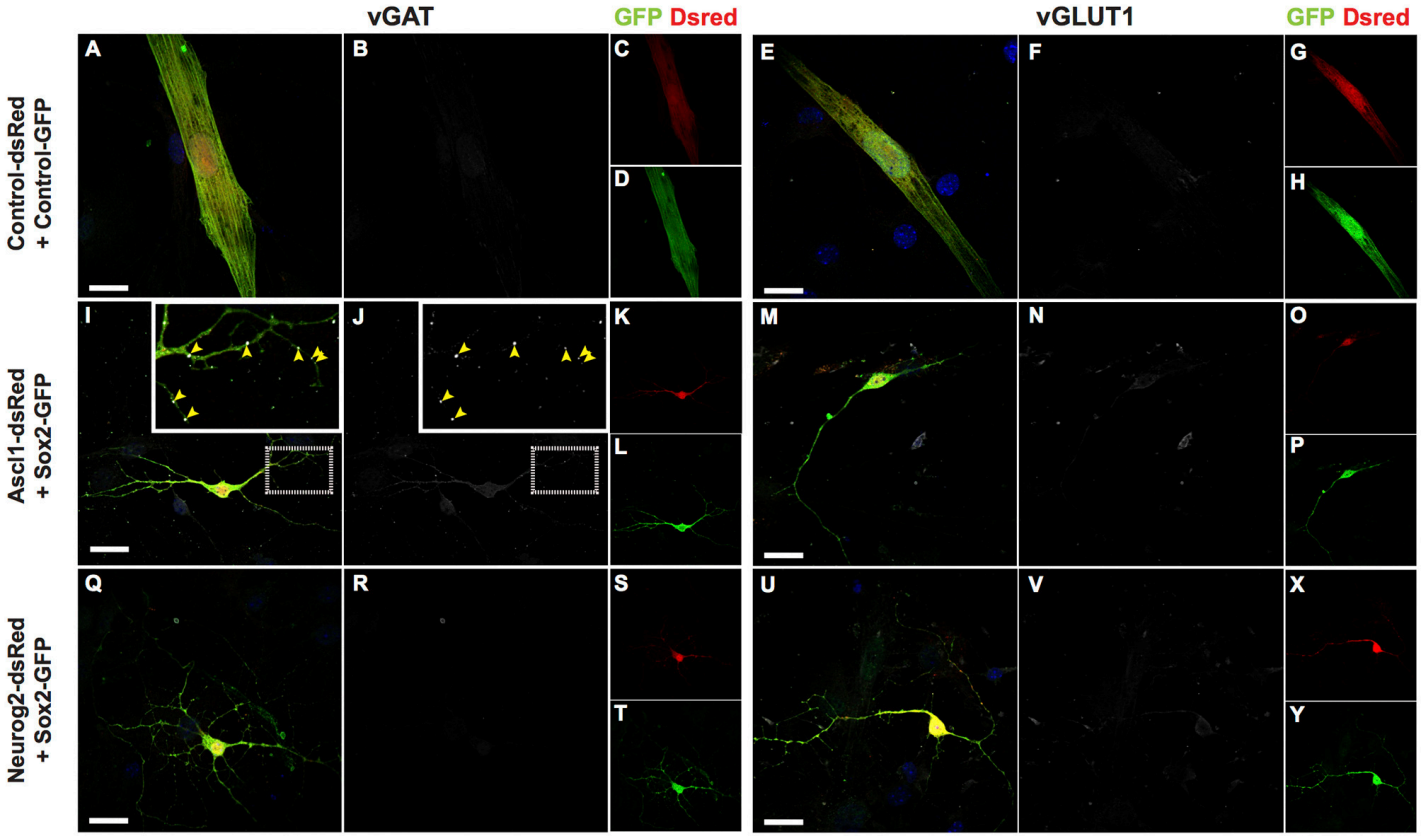
Protein expression of vesicular transporters of lineage reprogrammed hUCMSC-derived iNs. Immunostaining for DSRED (red), GFP (green), vGAT (white; left panel) or vGLUT1 (white; right panel) and DAPI (blue), 15 days post transfection (dpt). Scale bar represents 20μm. **(A–H)** hUCMSC transfected with control plasmids encoding only reporter proteins GFP and DSRED. Note that cells displayed classical mesenchymal cell morphologies and did not express neither vGAT or vGLUT1. **(I–P)** hUCMSC transfected with *Sox2* and *Ascl1*. **(Q–Y)** hUCMSC transfected with *Sox2* and *Neurog2*. **(I,J)** Observe the expression of the vesicular GABA transporter (VGAT) in *Sox2/Ascl1*-derived iN (yellow arrows). The inset shows a high magnification view of the boxed area.

To further evaluate the neurochemical phenotypes of hUCMSC-derived iNs, we investigated the expression of SLC17A7 (Vesicular Glutamate Transporter 1 or VGLUT1) and SLC32A1 (GABA Vesicular Transporter or VGAT) using immunocytochemistry (Figure 7). We observed that only a few *Sox2/Ascl1*-derived iNs showed expression of VGAT fifteen days after reprogramming (Figures 7I–L), whereas most of the hUCMSC-derived iNs did not express any of these markers days after (Figures 7M–Y). Although iNs expressed MAP2 15 days after transfection with proneural genes (Figure 1), expression of vesicular neurotransmitter transporters is likely to occur at later stages of neuronal differentiation. Further analyses and immunostaining for other vesicular transporters isoforms are necessary to confirm the phenotypes of hUCMSCs-derived iNs.

## Discussion

Direct lineage reprogramming of human somatic cells into neurons is a promising strategy to advance cell-based therapies to treat neurological disorders, as well as to study the basic mechanisms of neuronal differentiation. In this work, we further expand the list of cells suitable for direct lineage reprogramming using transcription factors. More importantly, we show that combined expression of either *Neurog2*/*Sox2* or *Ascl1*/*Sox2* is sufficient to convert human MSCs into iNs displaying electrophysiological properties typical of neuronal cells. Finally, we show that these two combinations of transcription factors may elicit diverse and non-exclusive neuronal phenotypes in reprogrammed cells.

Human MSCs are versatile cells, capable of differentiation into adipocytes, chondrocytes, and osteoblasts (Horwitz et al., 2005; Dominici et al., 2006). This potential, combined with the fact that hMSC can be easily isolated from adult donors, has encouraged researchers to further exploit the versatility of reprogramming MSCs to other lineages, such as muscle and neural cells for therapeutic purposes (Fan et al., 2011; Kwon et al., 2016). However, the capacity to convert MSCs into fully functional neurons using extrinsic signals remains a matter of intense debate.

Here, we show that forced expression of *Ascl1, Neurog2*, or *Sox2* alone is sufficient to convert hUCMSCs into iNs expressing key neuronal proteins and exhibiting electrophysiological properties of mature neurons. Importantly, combination of *Neurog2* or *Ascl1* with *Sox2* significantly increases the rate of hUCMSC conversion into iNs (up to 35% with *Sox2/Neurog2* and 49% with *Sox2/Ascl1*). This efficiency is similar to the conversion of human pericytes into iNs using *Sox2/Ascl1* (Karow et al., 2012) and significantly higher than the conversion rate of human fibroblasts into iNs using *Ascl1* or *Neurog2* alone (Chanda et al., 2014; Gascón et al., 2016) or the combination *Ascl1/Brn2/Myt1* (Caiazzo et al., 2011; Pang et al., 2011; Wapinski et al., 2013). However, the latter can be increased by using micro-RNAs, co-expression of *Bcl-2* and small molecule treatment (Yoo et al., 2011; Ladewig et al., 2012; Gascón et al., 2016).

Single expression of *Ascl1* is sufficient to convert other human somatic cells into iNs (Chanda et al., 2014). This potential of *Ascl1* is attributed to its ability to recognize and bind to the regulatory elements of its target genes even when they are nucleosome-bound (Wapinski et al., 2017). In contrast, the same pioneering activity has not been shown to *Neurog2*, which is believed to bind exclusively to accessible regulatory elements within the genome. This could help to explain the prominent potential of *Neurog2* to lineage-reprogram astrocytes (Berninger et al., 2007; Heinrich et al., 2010; Chouchane et al., 2017) and pluripotent stem cells (Zhang et al., 2013) in comparison to mouse embryonic fibroblasts into iNs (Gascón et al., 2016).

Our results indicate that a fraction of hUCMSCs (1–2%) has an epigenetic state compatible with the binding of NEUROG2 to regulatory elements of neuronal genes, allowing for the conversion into iNs. However, combination with *Sox2*, which has a well-known role in chromatin modification of Neurog2-target genes (Amador-Arjona et al., 2015), largely increases the efficiency of neuronal conversion mediated by *Neurog2* (~35%). Similarly, combination with *Sox2* increases the percentage of hUCMSCs converted by *Ascl1* into iNs by an order of magnitude. These observations suggest that key regulatory elements of neuronal genes identified by ASCL1 and NEUROG2 are not accessible in the vast majority of hUCMSCs cultured under the conditions described in this study.

In addition to the low frequency of neuronal conversion elicited in hUCMSC by forced expression of *Ascl1* or *Neurog2* alone, iNs also display electrophysiological properties less robust compared to iNs generated using *Sox2/Ascl1* or *Sox2/Neurog2*. In fact, the action potential of iNs reprogrammed with a single TF has a smaller amplitude and a shorter half-width as compared to iNs reprogrammed with 2 TFs (*Sox2/Ascl1* or *Sox2/Neurog2*), indicating that the latter express a more complete set of ion channels. It is possible that these differences represent a delay in the maturation of single-TF iNs. Alternatively, the combination of *Sox2/Ascl1* or *Sox2/Neurog2* may be necessary to induce the complete transcriptional cascade required for thorough neuronal maturation. Calcium transients are implicated in distinct aspects of neuronal differentiation by regulating neurotransmitter phenotype, dendritic morphology, and axonal growth and guidance (Rosenberg and Spitzer, 2011). While control-transfected hUCMSCs never displayed fast calcium transients, both *Sox2/Ascl1*- and *Sox2/Neurog2*-iNs showed spontaneous fast calcium transients, indicative of synaptic activity (Bonifazi et al., 2009). Likewise, the calcium transients of primary neurons and iNs are synchronized, suggesting that these cells are synaptically connected. Together, these findings suggest that hUCMSCs are lineage-converted into iNs capable of firing action potentials and establishing pre- and post-synaptic compartments.

The cellular and molecular mechanisms of direct lineage reprogramming remain largely unknown. It has been reported that the metabolic state is particularly important in direct neuronal reprogramming of somatic cells into iNs. Accordingly, co-expression of *Bcl2/Neurog2* or *Bcl2/Ascl1* greatly enhances the conversion efficiency of astrocytes into iNs by inhibiting lipid peroxidation, consistent with a caspase-independent role. Similarly, co-expression of *Bcl-2* alongside *Ascl1* improves the rates of lineage conversion of mouse embryonic fibroblast into iNs, demonstrating that the metabolic shift is necessary to support survival of lineage-converted iNs (Gascón et al., 2016). Our data suggest that mouse astrocytes and hippocampal neurons may contribute to enhance hUCMSCs survival during lineage conversion. Future experiments should elucidate whether Bcl2 co-expression or small molecules treatment would allow for the conversion of *Sox2/Neurog2*- or *Sox2/Ascl1*-iNs from hUCMSCs even in the absence of co-cultured cells.

Despite the large number of studies showing the conversion of human somatic cells into iNs, it remains largely unknown what is the phenotype of reprogrammed neurons (Ambasudhan et al., 2011; Pang et al., 2011; Son et al., 2011; Karow et al., 2012; Chanda et al., 2014; Hu et al., 2015). Moreover, it is still unclear whether different TFs could induce particular neuronal fates in lineage-converted cells. Here, we show that lineage-reprogrammed hUCMSCs generate iNs expressing genes associated with the acquisition of diverse neurotransmitter identities, regardless of the use of *Sox2/Ascl1* or *Sox2/Neurog2*. These different combinations of TFs can regulate similar sets of genes, suggesting that *Sox2/Ascl1* and *Sox2/Neurog2* are not sufficient to drive unambiguous neurotransmitter identities in hUCMSCs-derived iNs. However, the expression of genes associated with a specific neuronal phenotype is only an indication of the possible phenotype of the iNs. Future experiments using electrophysiological and pharmacological techniques are necessary to confirm the phenotypes of hUCMSCs-derived iNs.

According to the notion that *Neurog2* and *Ascl1* may be sufficient to induce a pro-neuronal program during somatic cell lineage reprogramming but not be sufficient to determine a specific phenotype of the iN, studies of the developing central nervous system reveal that those TFs may be associated with diverse neuronal phenotypes. For instance, while in the telencephalon, *Neurog2* plays important roles for the specification of glutamatergic neurons (Schuurmans and Guillemot, 2002). Progenitors in the cerebellum and spinal cord express *Neurog2* generate GABAergic and cholinergic neurons, respectively (Bertrand et al., 2002). Similarly, progenitors expressing *Ascl1* contribute to different neuronal lineages in the cerebral cortex, cerebellum, and retina (Chouchane and Costa, 2018). Most protocols aiming at obtaining fibroblast-derived iNs with a particular phenotype through direct lineage reprogramming require the use of several TFs (Victor et al., 2014; Blanchard et al., 2015).

Expression of either Ascl1 and Neurog2 in cortical astrocytes leads to the activation of transcriptional networks with only a small subset of shared target genes (Masserdotti et al., 2015), which could partly explain the role of those TFs in instructing different iNs phenotypes (Berninger et al., 2007; Heinrich et al., 2010). However, co-expression of Ascl1, Myt1L, and Brn2 induces a glutamatergic neuronal fate in fibroblast-derived iNs (Vierbuchen et al., 2010), whereas Neurog2 drives motor neuron differentiation associated with forskolin and dorsomorphin treatments in the same cells (Liu et al., 2013), suggesting that the fate-specification of iNs is not only dependent on the TF used. Recent work in our laboratory using direct lineage reprogramming of mouse astrocytes isolated from different brain regions further supports the versatile roles of *Neurog2* and *Ascl1* to affect the phenotypes of iNs (Chouchane et al., 2017). While cerebral cortex astrocytes reprogrammed into iNs with *Neurog2* adopt mostly a glutamatergic fate, cerebellum astrocyte-derived iNs show GABAergic phenotypes. Taken together, these data indicate that the cell of origin with its specific epigenetic landscape can influence the final fate of iNs.

A comprehensive understanding of the molecular mechanisms involved in the acquisition of particular neurochemical phenotypes will greatly improve the protocols for lineage reprogramming of human somatic cells into iNs, allowing for the generation of homogeneous neuronal populations that could be later used in cell-based therapies.

## Author contributions

All authors reviewed the manuscript. JAMA contributed to design, performed most of the experiments, analyzed the data, discussed the results, and wrote the manuscript. DAC performed isolation and characterization of hUCMSC. SRBM assisted and provided financial support with isolation and characterization of hUCMSC. RNL performed electrophysiology experiments. MMH analyzed electrophysiology experiments data, discussed the results, and helped writing the manuscript. DCFG performed qPCR experiments. DM-C analyzed the single cell qPCR data. MRC provided financial support, directed the project, conceived the experiment, analyzed data, discussed the results, and wrote the manuscript.

### Conflict of interest statement

The authors declare that the research was conducted in the absence of any commercial or financial relationships that could be construed as a potential conflict of interest.
