# Supplementary material for: Direct Reprogramming of Adult Human Somatic Stem Cells Into Functional Neurons Using Sox2, Ascl1, and Neurog2
Source: Front Cell Neurosci. 2018 Jun 8;12:155. doi: 10.3389/fncel.2018.00155 (PMC6003093; doi:10.3389/fncel.2018.00155)
Supplement: Supplementary file 3 [file Data_Sheet_1.DOCX]

Supplementary Material

Direct reprogramming of adult human somatic stem cells into functional neurons using *Sox2*, *Ascl1* and *Neurog2*

Jessica Alves de Medeiros Araujo, Markus M. Hilscher, Diego Marques-Coelho, Daiane Cristina Ferreira Golbert, Deborah Afonso Cornelio, Silvia Regina Batistuzzo de Medeiros, Richardson Naves Leão, and Marcos R. Costa^*^

*** Correspondence:** Marcos R. Costa: mrcosta@neuro.ufrn.br

# Supplementary Figures


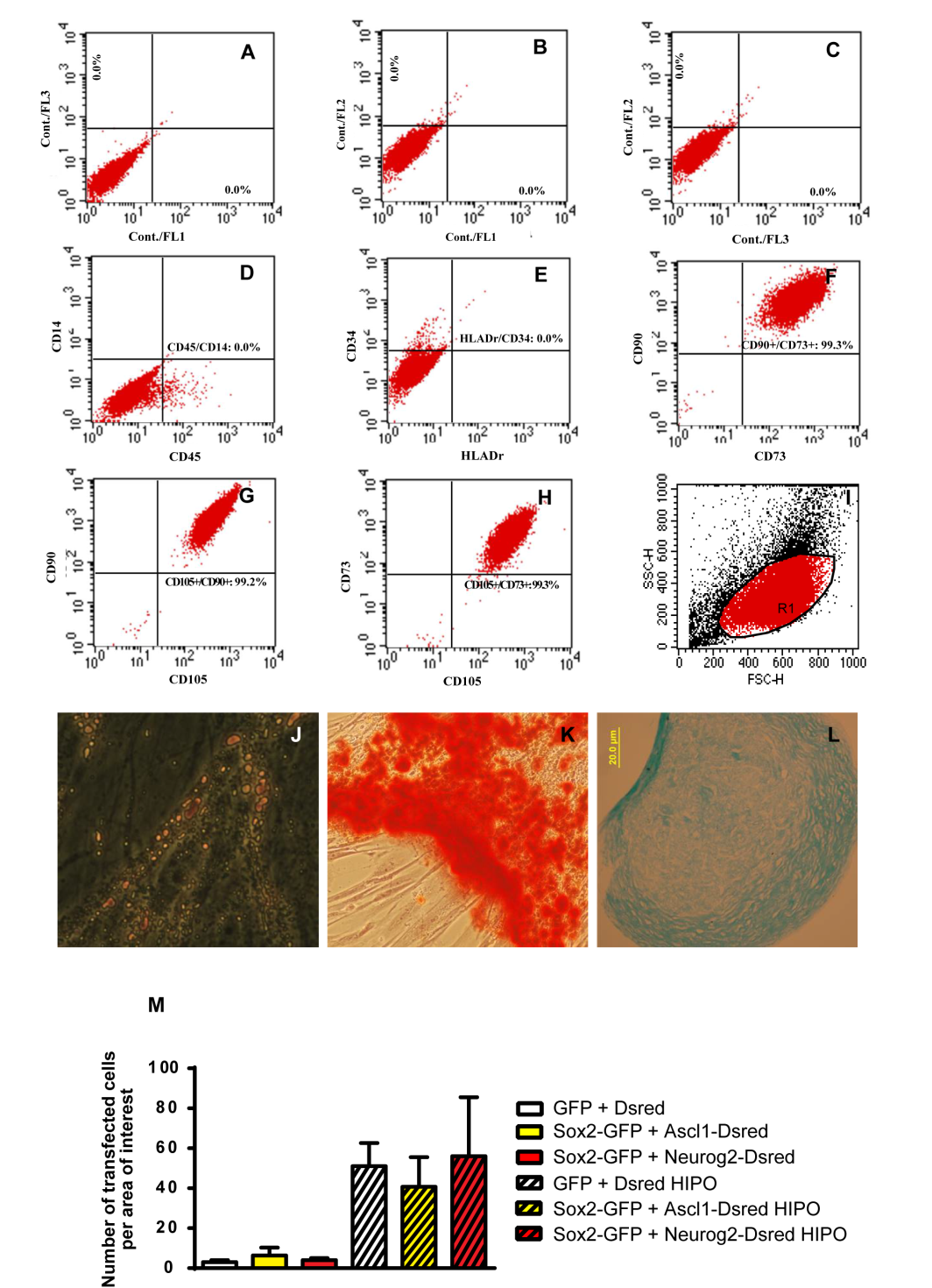


**Supplementary Figure 1.** Characterization of MSCs isolated from the human Wharton's jelly umbilical cord. (A-I) Representative plots expression of surface markers from human mesenchymal stem cell. (A-C) Negative controls using MSCs labeled with isotype control antibodies (Fl1-FITC, Fl2-PE-Cy5 and Fl3-PE). (D-I) Representative plots showing the expression of surface markers from human mesenchymal stem cell (CD105FITC, CD90PE-Cy5, CD73PE, CD34PE, HLA-DRFITC, CD45FITC, and CD14PE). Note the absence of CD14, CD34, HLA-DR, and CD45 expression D-E) and the positive expression for antigens CD90, CD73 and CD105 (F-H). (I) Forward scatter and side scatter measurements showing the complexity and size of MSCs (gate R1). (J-L) Differentiation potential of mesenchymal stem cells isolated from the human Wharton's jelly umbilical cord. (J) Adipogenic differentiation. Oil Red O staining of mesenchymal stem cells cultured for 21 days in adipogenic induction medium. (K) Osteogenic differentiation. Alizarin red staining of hMSCs cultured for 21 days in osteogenic induction medium. (L) Chondrogenic differentiation. Alcian Blue staining of paraffin-embedded sections of hMSCs cultured in pellet with chondrogenic induction medium.


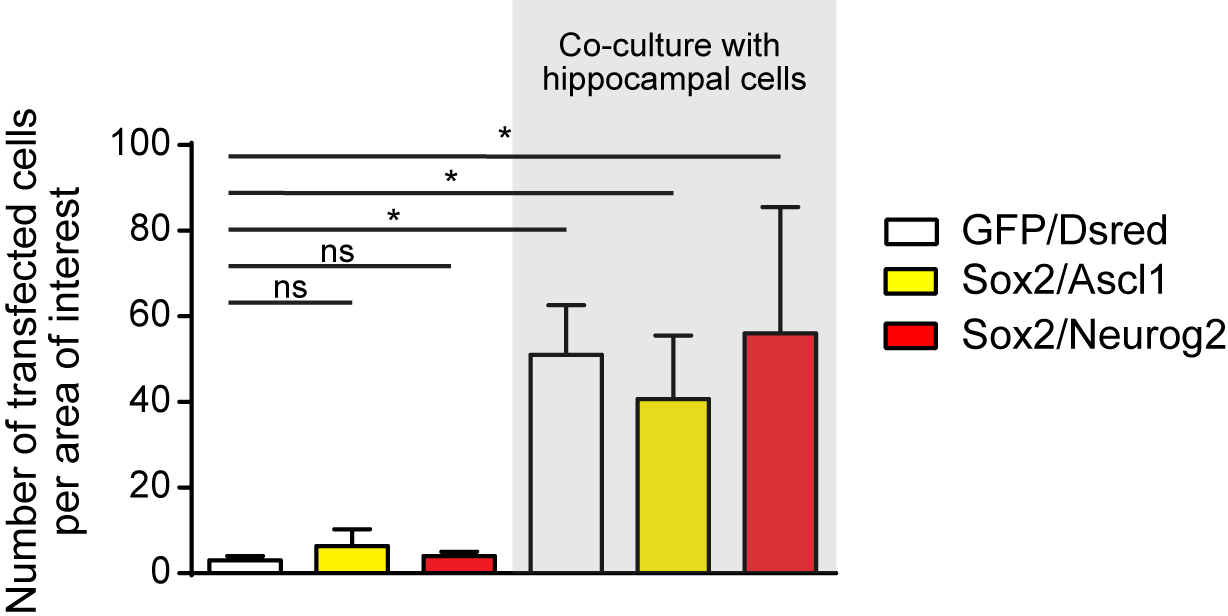


**Supplementary Figure 2.** Number of transfected hUCMSCs per area of interest 15 days after transfection. Histogram shows the total number of hUCMSCs transduced cells per area of interest. Data are presented as mean ± s.e.m. from three independent experiments. ANOVA followed by Dunn’s post hoc test, *p<0.05; no statistically significant difference (n.s.).

**
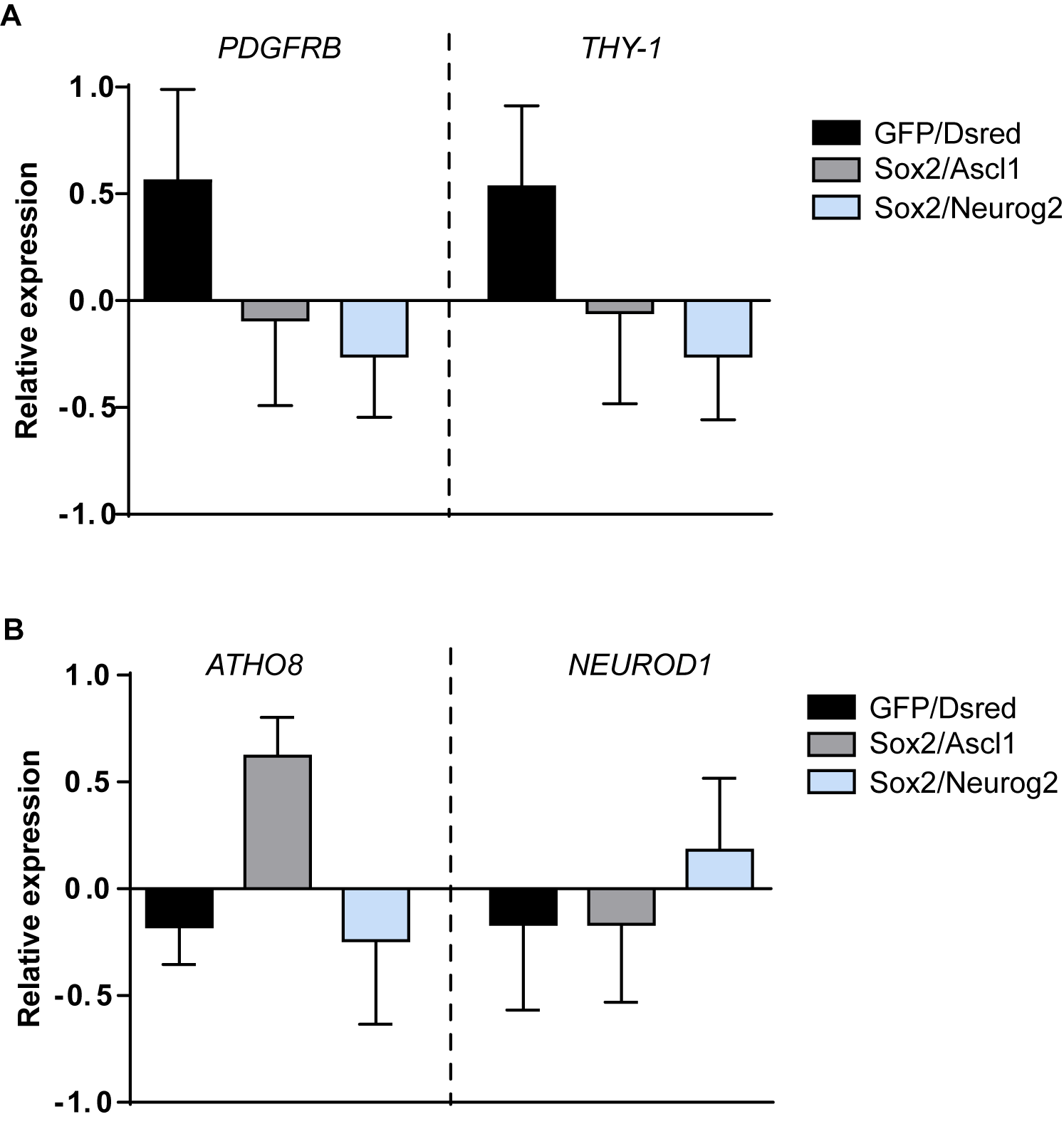
**

**Supplementary Figure 3.** Relative expression of genes normally expressed in hMSCs and neurons. Histograms show relative expression of Platelet-derived growth factor receptor, beta polypeptide (*PDGFRB*), Thy-1 cell surface antigen (*THY-1*), Atonal homolog 8 (*ATOH8*), and Neurogenic differentiation 1 (*NEUROD1*) in hUCMSCs after expression of pro-neural genes and plasmid controls.

# Supplementary Movies

**Supplementary Movie 1.** Human MSC lineage-converted iN with *Sox2* and *Neurog2* showed fast calcium transients. Movie shows 1200 frames taken with 10ms exposure time and no interval. Spontaneous calcium transients in hMSC reprogrammed with *Sox2* and *Neurog2* in the presence of hippocampal neurons. The imaged field is the same showed in figure 5 D and E.

**Supplementary Movie 2.** Human MSC lineage-converted iN with *Sox2* and *Ascl1* showed fast calcium transients. Movie shows 1200 frames taken with 10ms exposure time and no interval. Spontaneous calcium transients in hMSC reprogrammed with *Sox2* and *Neurog2* in the presence of hippocampal neurons. The imaged field is the same showed in figure 5 G and H.
